# EGCG Enhances the Chemosensitivity of Colorectal Cancer to Irinotecan through GRP78-MediatedEndoplasmic Reticulum Stress

**DOI:** 10.1155/2022/7099589

**Published:** 2022-09-13

**Authors:** Wenbing Wu, Hui Gou, Bin Xiang, Ruiman Geng, Jingying Dong, Xiaolong Yang, Dan Chen, Rongyang Dai, Lihong Chen, Ji Liu

**Affiliations:** ^1^Department of Biochemistry and Molecular Biology, West China School of Basic Medical Sciences and Forensic Medicine, Sichuan University, Chengdu 610041, China; ^2^Department of Biochemistry and Molecular Biology, School of Basic Medical Sciences, Southwest Medical University, Luzhou 646000, China; ^3^Department of Pharmacy, The Affiliated Hospital of Southwest Medical University, Luzhou 646000, China; ^4^Patent Examination Cooperation Sichuan Center of the Patent Office, China National Intellectual Property Administration, Chengdu 610041, China

## Abstract

This study aimed to explore the role of GRP78-mediated endoplasmic reticulum stress (ERS) in the synergistic inhibition of colorectal cancer by epigallocatechin-3-gallate (EGCG) and irinotecan (IRI). Findings showed that EGCG alone or in combination with irinotecan can significantly promote intracellular GRP78 protein expression, reduce mitochondrial membrane potential and intracellular ROS in RKO and HCT 116 cells, and induce cell apoptosis. In addition, glucose regulatory protein 78 kDa (GRP78) is significantly over-expressed in both colorectal cancer (CRC) tumor specimens and mouse xenografts. The inhibition of GRP78 by small interfering RNA led to the decrease of the sensitivity of CRC cells to the drug combination, while the overexpression of it by plasmid significantly increased the apoptosis of cells after the drug combination. The experimental results in the mouse xenografts model showed that the combination of EGCG and irinotecan could inhibit the growth of subcutaneous tumors of HCT116 cells better than the two drugs alone. EGCG can induce GRP78-mediated endoplasmic reticulum stress and enhance the chemo-sensitivity of colorectal cancer cells when coadministered with irinotecan.

## 1. Introduction

Colorectal cancer (CRC) is one of the top three malignant tumors in the world in terms of morbidity and mortality. The most common of these are adenocarcinomas, including colon adenocarcinoma (COAD) and rectum adenocarcinoma (READ), which account for 95% to 98% of all colorectal cancer cases. According to the statistics of GLOBOCAN provided by the International Agency for Research on Cancer of the World Health Organization in 2020, the number of new cases of CRC exceeded 1.9 million, accounting for 10% of the total number of cases, and the number of new deaths exceeded 935,000, accounting for 9.4% of the total number of cases, ranking 3rd and 2nd respectively [1]. In recent years, the incidence and mortality of colorectal cancer in China have maintained an increasing trend. According to the 2018 China Cancer Statistics Report, the incidence and mortality of colorectal cancer in China rank third and fifth among all malignant tumors, respectively. China has become the country with the largest number of new cases and death cases of CRC every year all over the world, which has seriously affected and threatened the health of the Chinese [2].

Although early screening and surgery are the primary means of prevention and treatment of colorectal cancer, chemotherapy remains a necessary systemic treatment for metastatic and recurrent CRC. Currently, the main chemotherapy drugs for CRC include fluorouracil, irinotecan, oxaliplatin, capecitabine, trefloxuridine, and raltitrexed. Irinotecan, a semi-synthetic camptothecin derivative, is a specific inhibitor of DNA topoisomerase I. Irinotecan and its active metabolite SN-38 can block DNA replication by binding to topoisomerase I-DNA complex, causing DNA double-strand break and apoptosis. However, the chemotherapy resistance and dose-limiting toxicity of irinotecan restrict its clinical application, such as drug resistance in patients with advanced non-small cell lung cancer (NSCLC) associated with ATP binding cassette subfamily G member 2 (ABCG2), breast cancer drug resistance induced by regulation of cell cycle and DNA repair activity [3], delayed-onset diarrhea and neutropenia [4]. Therefore, new and safe strategies are needed to improve the efficacy of irinotecan, increase drug sensitivity, and reduce toxic side effects.

Green tea is one of the most popular beverages. Epidemiological surveys have shown that drinking a large amount of green tea can reduce the risk of many chronic diseases, such as diabetes, cardiovascular disease, and various cancers [5]. The health benefits of green tea are mainly attributed to its main bioactive component catechin, in which EGCG is the most abundant component [6]. EGCG has strong antioxidant and free radical scavenging abilities due to the existence of multiple phenolic hydroxyl groups in its structure [7]. In recent decades, the anti-tumor activity of EGCG has gradually attracted the attention of scientists [8], especially its application in the prevention and treatment of gastrointestinal tumors such as gastric cancer and colorectal cancer. EGCG and other polyphenols have attracted extensive attention due to their low side effects and wide availability [9].

In previous studies, we found that EGCG enhanced irinotecan-induced DNA damage and autophagy in CRC cells [10]. It has been previously reported that endoplasmic reticulum stress might regulate the repair of DNA damage. The ERS inhibitor taurodeoxycholic acid (TUDCA) suppressed the DNA damage marker *γ*-H2AX of the late porcine embryo [11], and ERS inducers (tunicamycin and glucose deprivation) led to the selective degradation of DNA repair protein RAD51, thereby inhibiting the DNA double-strand break repair of lung cancer cell [12]]. Due to the rapid proliferation of cancer cells and their easy exposure to a low-nutrient, low-vascularization and hypoxic environment, endoplasmic reticulum stress-related proteins, such as GRP78, activating transcription factor 6 (activating transcription factor 6, ATF6), inositol-requiring enzyme 1 (IRE1), protein kinase RNA-like endoplasmic reticulum kinase (protein kinase RNA-like ER kinase, PERK) are overexpressed in many types of tumors. In response to early endoplasmic reticulum stress, cells initiate the UPR, which promotes protein folding and degrades unfolded proteins as an adaptive survival pathway [13]. Under ER stress, GRP78 dissociates from the luminal region of three ER sensors (IRE1, PERK, and ATF6). Three ER-localized UPR transmembrane signal transducers detect the accumulation of unfolded proteins that initiate restoration and maintenance of endoplasmic reticulum homeostasis. However, if the endoplasmic reticulum is stressed for a long time and the unfolded protein fails to form a protein in the correct conformation, the UPR switches to the apoptotic pathway and induces apoptosis [14]. Studies have shown that EGCG can selectively induce disturbance of Ca^2+^ homeostasis in mesothelioma cells and promote endoplasmic reticulum stress [15], thereby triggering apoptosis and even necrosis [16].

In this study, we confirmed that EGCG alone or in combination with irinotecan could up-regulate the GRP78, activate ERS of colorectal cancer cells, reduce intracellular reactive oxygen species and mitochondrial membrane potential, and induce apoptosis. At the same time, the mouse xenograft experiment also confirmed the synergistic effect of EGCG and irinotecan on ERS and tumor cell apoptosis.

## 2. Materials and Methods

### 2.1. Materials

RKO cells were purchased from Procell (Wuhan, China), and HCT116 cells were purchased from NuoHe Bio-Tech (Chengdu, China). All the cell lines used have been authenticated, and mycoplasma testing has been carried out. EGCG, irinotecan, and JC-1 were purchased from CSN pharm (Shanghai, China), Bradford protein concentration assay kit (detergent-compatible type), and reactive oxygen species detection kit were purchased from Beyotime (Shanghai, China). GAPDH, ACTIN, Bax, Bcl-2, PARP, GRP78, Ki67, and cleaved-caspase3 antibodies were purchased from Proteintech (Wuhan, China). The GRP78 overexpression plasmid was designed by Genechem (Shanghai, China), and the GRP78 siRNA was provided by Tsingke (Beijing, China). Lipofectamine™ 2000 Transfection Reagent was purchased from Invitrogen (California, USA). Trizol reagent and HiScript® III reverse transcription kit was purchased from Vazyme (Nanjing, China). FastQuant RT Kit (With gDNase) reverse transcription kit was purchased from Tiangen (Beijing, China), and TB Green Premix Ex Taq II (Tli RNaseH Plus) was purchased from Takara (Dalian, China). Annexin V-FITC apoptosis assay kit was purchased from Absin (Shanghai, China). SABC (mouse IgG)-POD kit was provided by Solarbio (Beijing, China).

### 2.2. Cell Culture

The method mainly refers to the book “Human Cell Culture Protocols” by Philippeos et al. [17]. Briefly, cells were taken out of the liquid nitrogen and thawed at 37°C, then centrifuged at 1000 rpm for 3 minutes. After being added with 1640 medium containing 10% serum, cells were cultured in a Forma Series II Water Jacket CO_2_ Incubator (Thermo Fisher Scientific Instruments, Ohio, USA) at 37°C in a 5% CO_2_ atmosphere.

### 2.3. Mitochondrial Membrane Potential Detection

The drug-treated cells in the 12-well plate were washed three times with pre-cooled PBS. 10 *µ*g/mL of JC-1 (5, 5′, 6, 6′-Tetrachloro-1, 1′, 3, 3′-tetraethyl-imidacarbocyanine iodide) solution was added and incubated at 37°C for 20 min in the dark. Cells were washed twice with PBS to detect the red and green fluorescence by BD FACS flow cytometer (BD Biosciences, New Jersey, USA) or BD Accuri C6 flow cytometer (BD Biosciences, New Jersey, USA).

### 2.4. ROS Analysis

The intracellular ROS production was detected by the DCFH-DA method according to the manufacturer's instructions. Briefly, 2′, 7′-Dichlorofluorescin Diacetate (DCFH-DA) was diluted with 1640 serum-free medium (1 : 1000) and added to the culture plate, then incubated at 37°C for 20 minutes. Followed by washing three times with PBS and trypsinization, the cells were collected in Eppendorf tubes. The green fluorescence was detected by flow cytometry (BD Biosciences, New Jersey, USA).

### 2.5. Transient Transfection of GRP78

When the cell confluence reaches 70–90%, the following operations are performed. Taking a 12-well plate as an example, 2 *μ*L of GRP78-expressing plasmid DNA or control plasmid DNA (400 ng/*μ*L) mixed with 25 *μ*L serum-free medium, and stand still for 5 min at room temperature. Meanwhile, 2 *μ*L lipo2000 and 25 *μ*L serum-free medium were mixed too. The two solutions were mixed and added to the cell culture for another 24 hours. The transfection efficiency was determined by qPCR and western blot. For siRNA transfection, mixed 1 *μ*L siGRP78/control siNC (20 pM), or 1 *μ*L of lipo2000 with 25 *μ*L of serum-free medium, respectively. Other operations are the same as above.

### 2.6. RNA Extraction and Real-Time PCR

The RNA was extracted from the transfected cells in the 6-well plate according to the Trizol method [18], and the total cell RNA was reverse transcribed into cDNA according to the kit instructions. Using cDNA as a template and GAPDH as an internal reference gene, qPCR amplification was performed according to the gene amplification instructions to detect the expression of the GRP78 gene. Briefly, the primer sequences, template cDNA, TB Green Mix, and RNase-free water in Table 1 were mixed to form an amplification system. The annealing temperature was 60°C for PCR amplification, and the 2^−△△Ct^ method was used for relative gene quantitative analysis.

### 2.7. Western Blot

After the drug-treated cells or animal tissues were lysed, the total cell protein was extracted, and the protein concentration was determined according to the Bradford method. Prepare 3% separating gel and 10% stacking gel according to the manufacturer's instructions, and use a microinjector to sample 30 *μ*g of protein for electrophoresis. The gel was taken out and transferred to an NC membrane, blocked with 0.05% TBST containing 5% skim milk for 1 h at room temperature, primary antibody (1 : 1000) was added, and incubated overnight at 4°C. After incubation with HRP-labeledanti-mouse or rabbit secondary antibody (1 : 5000) for 1 h at room temperature, the photograph was obtained by Universal Hood II Gel Imaging System (Bio-Rad Instruments, California, USA).

### 2.8. Cell Apoptosis Detection

The apoptosis was detected by Annexin V-FITC&PI double-staining, and the cells treated with drugs in a 12-well plate were operated according to the kit instructions. Briefly, the medium and trypsin digested cells were collected simultaneously and the supernatant was centrifuged. After the cells were washed with PBS and collected by centrifugation, the mixture of Annexin V-FITC and PI staining solution was added and kept from light for 15 minutes at room temperature. The green and red fluorescence were detected by BD FACS flow cytometry (BD Biosciences, New Jersey, USA).

### 2.9. Immunohistochemical Assay

The tumor tissue fixed with 10% formaldehyde for more than 48 h was taken out, washed with water, and slightly trimmed. After gradient alcohol dehydration, xylene transparency, and paraffin embedding, the tissue in the embedding block was cut into 4-5 *μ*m thick slices with a tissue slicer. According to the instructions of the IHC kit, the expression of ki67, GRP78, and Cleaved-caspase3 in tissues was detected by the immune and enzymatic reaction principle of “tissue antigen-GRP78 mouse primary antibody-biotin labeled secondary antibody-streptavidin coupled peroxidase-substrate DAB”. The photograph was obtained by DM2500 fluorescence microscope (Leica Microsystem, Wetzlar, Germany).

### 2.10. Detection of Cell Surface GRP78 by ELISA

The cells were inoculated in a 96-well plate, and after being treated with drugs for a fixed time, the cells were washed twice with PBS. Cells were fixed in 4% formaldehyde (PBS dilution) for 10 minutes, after washing 3 times with wash buffer (PBS containing 0.5 mM CaCl_2_, 1 mM MgCl_2_, and 0.1% Triton) the cells were blocked with 3% BSA (diluted in wash buffer) for 30 minutes. Cells were incubated with GRP78 antibody (1 : 500 dilutions in wash buffer containing 1% BSA) for 2 hours at room temperature and washed three times. Cells were incubated with 3, 3′, 5, 5′- tetramethylbenzidine (TMP) substrate for 10 minutes followed by reading in an iMark microplate reader (Bio-Rad Laboratories, California, USA) at 620 nm.

### 2.11. Tumor Formation Assay

Female nude mice (Balb/c; 6 weeks old; weight 18 ± 1 g) were purchased from Beijing Charles River. The concentration of HCT116 cells in the logarithmic phase was adjusted to 2.5 × 10^7^/mL, and the 200 *μ*L cell suspension was inoculated subcutaneously on the right dorsal side of the mouse. When the average tumor volume reached 100 mm^3^, animals were randomized into 4 groups (5 mice for each group), control (Control, normal saline, 1 time per day, ip), irinotecan (IRI, 4 mg/kg irinotecan, 2 times per week, ip), EGCG (EGCG, 5 mg/kg, 1 time per day, ip), and irinotecan in combination with EGCG (IRI + EGCG). The mental state and health condition of the nude mice were observed daily and weighed once a week. The long (a/mm) and short diameter (b/mm) of the tumor were measured with a vernier caliper. According to the formula of tumor volume (*V*/mm^3^): *V* = *a* × *b*^2^/2, the tumor volume was calculated. After continuous administration for four weeks, tumor tissues were harvested. One part of tumor tissues were stored at −80°C for qPCR and WB detection, and the other part was immersed in 10% formaldehyde for fixation and underwent IHC detection.

### 2.12. Data Analysis and Statistics

The data detected by flow cytometry were analyzed by FlowJo software, and the Average Optical Density of IHC results was calculated by ImageJ software. GraphPad Prism 7.0 was used for statistical analysis and diagram drawing. All experiments were repeated three times. The one-way analysis of variance (ANOVA) and multiple comparisons were used for statistical comparison between groups. The difference was considered significant if *P* < 0.05.

## 3. Results

### 3.1. EGCG Alone or in Combination with Irinotecan Aggravates Mitochondrial Dysfunction in CRC

**Figure 1:**
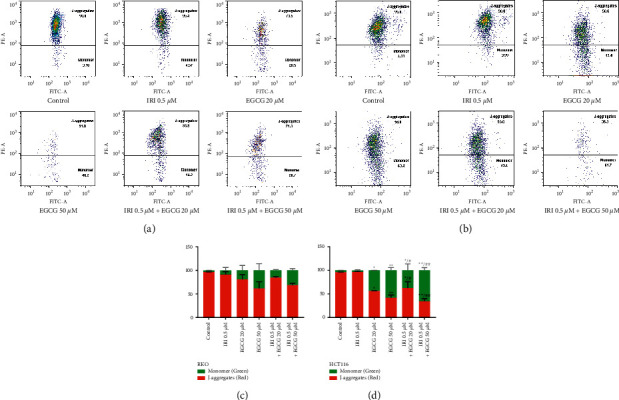
Effects of EGCG on MMP of irinotecan pretreated RKO and HCT116 cells. JC-1 staining was used to detect the conversion of RKO (a) and HCT116 (b) cells from red aggregates to green monomers, and the data were fitted with FlowJo software. (c, d) Statistical charts. ^*∗*^*P* < 0.05 and ^*∗∗*^*P* < 0.01(vs control group). ^#^*P* < 0.05 and ^##^*P* < 0.01 (vs IRI 0.5 *µ*M group).

In the early stage of apoptosis, mitochondrial membrane potential (MMP) will collapse. JC-1 would exhibit different fluorescence intensities due to different concentrations, and its degree of aggregation could reflect the surface potential of the mitochondrial membrane. Therefore, when the change of JC-1 from red fluorescence (J-aggregates) to green fluorescence (Monomer) was observed, it indicated that MMP was decreased and mitochondrial depolarization might induce early apoptosis [19]. As shown in Figure 1, JC-1 staining and flow cytometry results showed that EGCG increased the proportion of depolarized mitochondria from 3.18% (Control) to 14.2% (IRI 0.5 *μ*M + EGCG 20 *μ*M) and 28.7% (IRI 0.5 *μ*M + EGCG 50 *μ*M) in RKO cells when exposed to irinotecan for 24 hours. While the proportion rose up dramatically from 4.23% (Control) to 47.3% (IRI 0.5 *μ*M + EGCG 20 *μ*M) and 61.7% (IRI 0.5 *μ*M + EGCG 50 *μ*M) on HCT116 cells. Overall, these results indicated that EGCG could significantly reduce the mitochondrial membrane potential of irinotecan-pretreated colorectal cancer cells.

### 3.2. EGCG Alone and in Combination with Irinotecan Promote CRC Apoptosis

**Figure 2:**
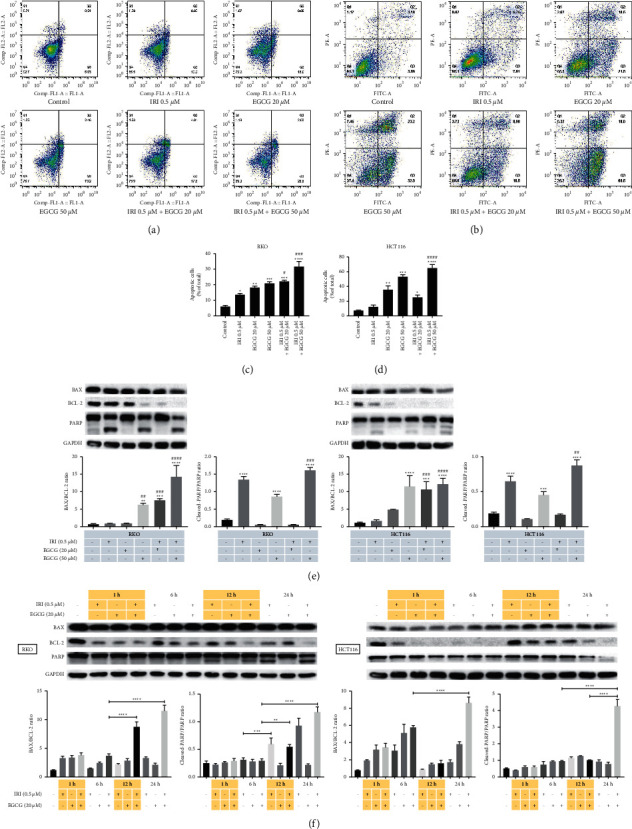
Effects of EGCG on apoptosis of irinotecan pretreated RKO and HCT116 cells. Annexin V-FITC&PI double staining was applied to determine the apoptosis rate of RKO (a) and HCT116 (b) cells and FlowJo software was used to fit the data (c, d). Apoptosis-related proteins with EGCG dose (e) and treatment time (f) were carried out by western blot. ^*∗*^*P* < 0.05, ^*∗∗*^*P* < 0.01, ^*∗∗∗*^*P* < 0.005, and ^*∗∗∗∗*^*P* < 0.001 (vs control group); ^#^*P* < 0.05, ^###^*P* < 0.005, and ^####^*P* < 0.001(vs IRI 0.5 *µ*M group).

Flow cytometry analysis of apoptosis showed that EGCG significantly increased the apoptosis of RKO and HCT116 colorectal cancer cells, and exerted a synergistic effect with irinotecan. EGCG significantly inhibited the expression of anti-apoptotic protein Bcl-2, but had little effect on the pro-apoptotic protein BAX, thus increasing the ratio of Bax to Bcl-2. In addition, cleavage of PARP increased with EGCG concentration and treatment time (Figure 2). These results demonstrated that EGCG promoted irinotecan apoptosis induction in a dose and time-dependent manner.

### 3.3. EGCG Alone and in Combination with Irinotecan Inhibit ROS Production in CRC

**Figure 3:**
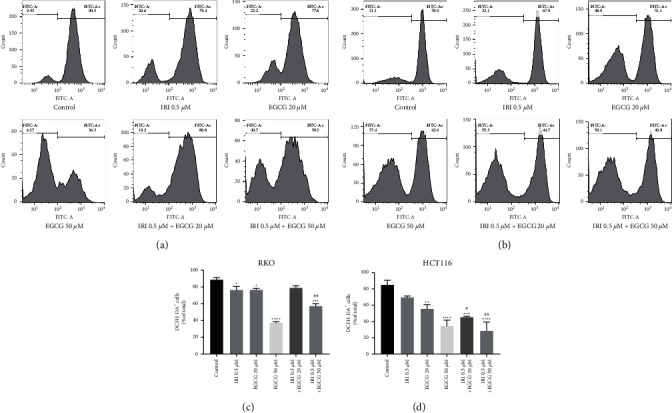
Effects of EGCG on ROS in RKO and HCT116 cells pretreated with irinotecan. DCFH-DA probe method was applied to determine ROS in RKO (a) and HCT116 (b) cells and FlowJo software was used to fit the data. (c, d) Statistical charts. ^*∗*^*P* < 0.05, ^*∗∗*^*P* < 0.01, ^*∗∗∗*^*P* < 0.005, and ^*∗∗∗∗*^*P* < 0.001 (vs control group); ^#^*P* < 0.05, ^##^*P* < 0.01, and ^###^*P* < 0.005 (vs 0.5 *µ*m IRI group).

Dichloro-dihydro-fluorescein diacetate (DCFH-DA), a non-fluorescent precursor of DCF, can be used as a probe for intracellular oxidative stress. DCFH-DA is extremely sensitive to changes in the intracellular redox state. Cytolactonase breaks down DCFH-DA at the diester bond and produces a relatively polar and cell membrane-impermeable product, H_2_DCF. This non-fluorescent molecule accumulates in the cells and is subsequently oxidized by the intracellular oxidant to produce the highly fluorescent product DCF, whereupon the redox state of the cells can be detected by detecting the changes in fluorescence [20]. The results in Figure 3 suggested that RKO and HCT116 cells had high endogenous ROS levels, while irinotecan or EGCG significantly reduced intracellular ROS concentrations. When the two drugs were combined, the scavenging effect on ROS was more significant and showed an EGCG dose-dependent manner. Together, these results provide important insights into that EGCG, either alone or in combination with irinotecan, could significantly inhibit ROS in CRC cells.

### 3.4. EGCG Mediates the Constitutive UPR of Colorectal Cancer Cells into Endoplasmic Reticulum Stress to Promote Apoptosis by Inducing the Accumulation of GRP78

**Figure 4:**
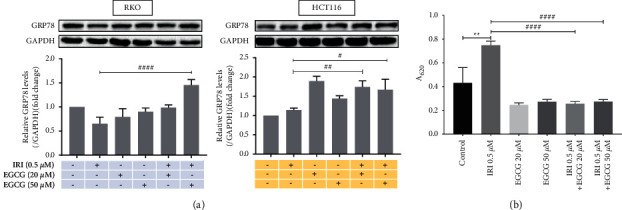
Effects of EGCG on GRP78 in RKO and HCT116 cells pretreated with irinotecan. GRP78 protein was detected by western blot (a), and GRP78 content in HCT116 cell membrane was detected by cell-surface ELISA (b). ^*∗∗*^*P* < 0.01 (vs Control group), ^#^*P* < 0.05, ^##^*P* < 0.01, ^###^*P* < 0.001 (vs 0.5 *μ*m IRI group).

EGCG can induce endoplasmic reticulum stress in mesothelioma cells through the GRP78/ATF4/CHOP axis [21]. In addition to up-regulation in cancer cells, GRP78 may also translocate from the endoplasmic reticulum to the cell membrane, where it mediates the transmission of extracellular signals as a membrane receptor, for example, as a receptor for *α*2-macroglobulin [22] or as a receptor/cofactor for GPI anchor signaling protein Cripto [23]. Therefore, the event of membrane translocation of GRP78 may serve as a potential therapeutic target for tumors. Cell surface enzyme-linked immunosorbent assay (ELISA) is developed based on early enzyme immunochemistry (EIH) and ELISA. It is a simple, rapid, and highly sensitive method for detecting cell surface molecules [24]. As shown in Figure 4, both colorectal cancer cell lines exhibited a certain degree of constitutive GRP78 expression, and irinotecan inhibited the expression of GRP78 in RKO cells. Compared with the irinotecan alone group, the combination of EGCG promoted intracellular GRP78 expression in RKO and HCT116 cells. HCT116 cell surface ELISA results showed that, compared with the irinotecan group, the content of cell surface GRP78 was decreased by nearly two-thirds in either the group with or without EGCG, indicating that EGCG could inhibit GRP78 membrane translocation induced by irinotecan. Therefore, we speculate that EGCG can promote the transformation of constitutive UPR of colorectal cancer cells into endoplasmic reticulum stress by increasing the accumulation of intracellular GRP78 and inhibiting its cell membrane translocation.

**Figure 5:**
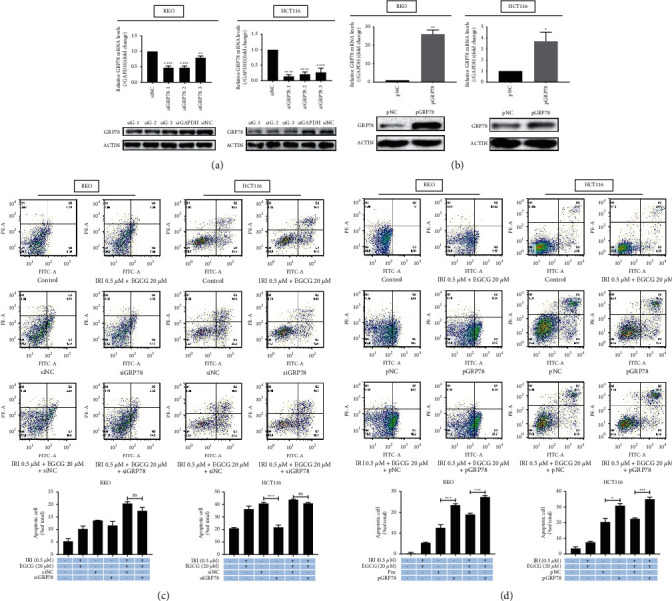
Effects of EGCG combined with irinotecan on GRP78-mediated ER stress and apoptosis in RKO and HCT116 cells. GRP78 mRNA and protein expression in RKO and HCT116 cells were detected by qPCR and WB after transfection of GRP78 overexpression plasmid (a) or siRNA (b) 24 h. Annexin V-FITC and PI double staining method was employed to determine the effect of GRP78 on drug combination-treated cell apoptosis, and FlowJo software was used to fit the data (c, d). siG-siGRP78, siGAPDH-positive control, siNC-negative siRNA control, pNC-vector control, pGRP78-GRP78 overexpression plasmid. ^*∗*^*P* < 0.05, ^*∗∗*^*P* < 0.01, ^*∗∗∗*^*P* < 0.005, and ^*∗∗∗∗*^*P* < 0.001; ns: not significant (vs corresponding NC group).

The GRP78 gene and protein expression in both cells was manipulated by transfection of the siRNA or overexpression plasmid of the GRP78 gene (Figures 5(a) and 5(b)). GRP78 is the main molecular chaperone of the endoplasmic reticulum and also the central sensor of cell stress. In some tumor cells, EGCG can regulate cell endoplasmic reticulum stress and apoptosis by targeting GRP78 [25, 26]. By knocking down or overexpressing GRP78 and flow cytometry analysis of apoptosis, we found that up-regulation of GRP78 by EGCG combined with irinotecan was an important cause of apoptosis. Knocking down GRP78 by siRNA alone had little effect on the apoptosis of RKO cells, but it significantly inhibited the apoptosis of HCT116 cells. Meanwhile, when EGCG and irinotecan were combined, the knock-down effect of GRP78 on apoptosis was not significant. However, when cells were transfected with GRP78 overexpression plasmid, apoptosis was also significantly increased (Table 1). The synergistic induction of irinotecan with EGCG on DNA damage in colorectal cancer cells is consistent with the results [9]. The promotion of GRP78 by EGCG is also the possible reason that the increase of cell ERS leads to the inhibition of DNA damage repair and eventually leads to apoptosis. Taken together, EGCG mediates the transition from constitutive UPR to pro-apoptotic endoplasmic reticulum stress in colorectal cancer cells by inducing GRP78 accumulation.

### 3.5. EGCG Alone or in Combination with Irinotecan Inhibits the Growth of Xenografts in Mice

**Figure 6:**
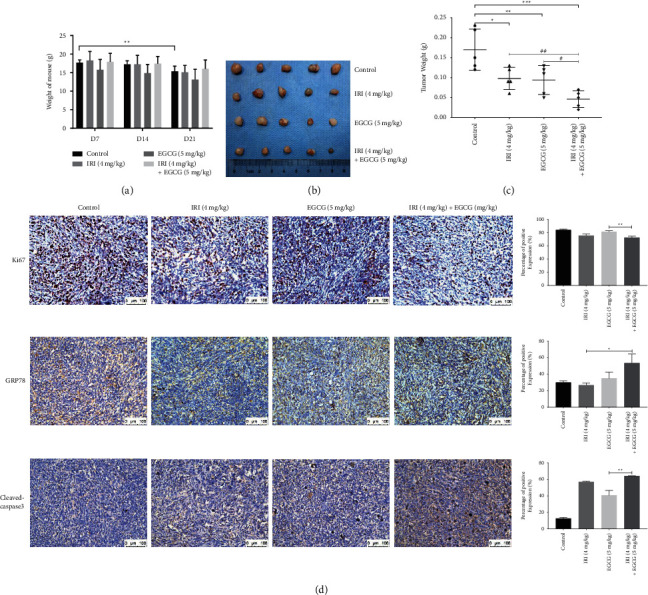
Effects of EGCG combined with irinotecan on the growth of subcutaneous colorectal tumor in balb/c nude mice. Changes in body weight (a) and tumor volume (b, c) of HCT116 cell xenograft mice treated with EGCG and/or irinotecan. (d) The expression of Ki67, GRP78, and cleaved-caspase3 was detected by IHC after taking out the mouse tumor. ^*∗*^*P* < 0.05, ^*∗∗*^*P* < 0.01, and ^*∗∗∗*^*P* < 0.005. ^#^*P* < 0.05, ^##^*P* < 0.01.

Two weeks after inoculation with HCT116 cells, Balb/c nude mice with an average tumor volume of 100 mm^3^ were started on dosing. During the treatment period, the overall state of the mice was good, and no significant adverse reactions were observed. Monitoring results showed no significant decrease in body weight among the dosing groups except for a significant decrease in the control group compared to the initial dosing (Figure 6(a)). In terms of appearance and morphology, the tumor mass in the control group was the largest, followed by the irinotecan group and EGCG group with no significant difference, and the combination group was the smallest (Figure 6(b)). The results of tumor mass measurement showed that compared with the control group, the masses of the other three groups were significantly reduced, and the combined group was the most significant. In addition, the combined doses were significantly less massive than the irinotecan or EGCG monotherapy groups (Figure 6(c)). The transverse section of the subcutaneous tumor in mice was gray and fish-like, and the boundary between the tumor and the surrounding tissues was clear. Tumor IHC results showed a significant decrease in the expression of cell proliferation marker Ki67 in the combination group, and a significant increase in GRP78 compared with irinotecan alone. The apoptotic marker cleaved-caspase3 was also significantly higher in the combination group than in the EGCG monotherapy group (Figure 6(d)). As shown in the *in vitro* experiments, these results indicated that EGCG also exerted a synergistic effect with irinotecan in inhibiting the growth of CRC in xenograft tumor model mice.

## 4. Discussion

Time-dependent resistance to chemotherapy and radiotherapy, as well as treatment interruption due to side effects, are major problems in cancer treatment. Therefore, the development of new strategies, including the combination of traditional therapies and bioactive dietary compounds, has aroused scientists' great interest in recent years. These new cancer treatment strategies have been demonstrated to exert additional or synergistic effects when combined with chemotherapy or radiotherapy and to minimize treatment-induced toxicity. In the previous studies, we demonstrated through cell proliferation, migration, and invasion that EGCG and irinotecan, a clinical chemotherapy drug for colorectal cancer, can exploit the synergistic anti-tumor effect on cells [10].

During respiratory oxidation, mitochondria can produce proton gradient differences on both sides of the mitochondrial membrane, which is the basis for the formation of mitochondrial membrane potential. As reported, under some external causes, cells will suffer from serious depletion of mitochondrial membrane potential, thus inducing apoptosis [27]. The cells treated with 50 µM EGCG for 0.5–24 hours in human glioblastoma cells [28], or 50–100 µM EGCG for 72 hours in epidermoid carcinoma and breast adenocarcinoma cells both observed a decrease in mitochondrial membrane potential [29]. Our results also confirmed that EGCG can reduce MMP and induce depolarization through a mitochondrial-dependent pathway, leading to increased apoptosis (Figures 1 and 2). Animal experiments confirmed that EGCG and irinotecan could exert a synergistic effect against subcutaneous tumors of colorectal cancer, and the tumor inhibition rate was higher with the combination of EGCG and irinotecan than with a single drug. Compared with the control group, the mice in the medication group did not lose significantly. IHC results showed that the expression levels of Cleaved-caspase3 were significantly increased in the combination group, while the expression of Ki67 was significantly decreased (Figure 6).

In cancer cells, reactive oxygen species are mainly produced by the high-speed metabolism of mitochondria, endoplasmic reticulum, and cell membrane. The increase of ROS in cancer cells may be related to a variety of mechanisms, such as the inactivation of tumor suppressor genes, activation of oncogenes, hypermetabolism, and mitochondrial dysfunction [30]. Due to abnormal regulation of redox homeostasis and possible stress adaptation, cancer cells can tolerate high levels of endogenous oxidative stress in vivo and in vitro. The cells' ability to actively produce and accumulate a large amount of ROS prevents them from suffering from the harmful effects of oxidative stress. Moreover, they may increase the stress adaptation ability of tumor cell populations by applying selective pressure, which may also be one of the reasons leading to tumor heterogeneity [31]. Different from the results obtained in some tumor cell experiments that EGCG functions as a pro-oxidant to increase the intracellular ROS concentration [32, 33], we have found that the constitutive ROS level in colorectal cancer cells is high, and EGCG can scavenge intracellular reactive oxygen species (Figure 3). Abnormal ROS may be a feature of cancer cells, while the ROS at high concentrations might be an indicator of drug resistance. It has been reported that gefitinib resistance is related to mitochondrial dysfunction and increased cellular ROS in lung cancer cells [34]. Therefore, it is speculated that EGCG may reduce the resistance of colorectal cancer cells to irinotecan by down-regulating cell ROS levels, but further research is required.

The endoplasmic reticulum is a dynamic tubular network responsible for the biosynthesis, folding, and transportation of protein, maintaining calcium homeostasis, and regulating many other cellular physiological processes. The extremely sensitive ER will lead to ERS and UPR activation due to blocked protein folding and maturation. Many endoplasmic reticulum members, such as GRP78, protein disulphide isomerase (PDI), calnexin, and calreticulin, play important roles in protein folding and preventing protein aggregation [35]. The interference of EGCG on UPR signal has been described in different studies. In non-cancer mouse retinal pigment epithelial cells, EGCG has been found to down-regulate the GRP78 and UPR signals [15]. However, UPR in cancer cells is usually different, in mouse liver cancer EGCG shows pro-apoptotic activity related to ERS induction [36]. The cell membrane surface GRP78 can be used as an extracellular receptor that binds to ligands such as alpha-2-macroglobulin(*α*2M) and T-cadherin, and activates signaling pathways related to cell survival, proliferation, or apoptosis [37]. By detecting the expression of GRP78 protein in colorectal cancer cells intracellularly and on the membrane surface, we found that EGCG could convert cellular constitutive UPR into endoplasmic reticulum stress and induce apoptosis by promoting the intracellular accumulation of GRP78 and reducing membrane content (Figures 4, 5(c), and 5(d)), which is consistent with the result of Simona [21]. Accidently, the combination of EGCG and irinotecan did not significantly inhibit apoptosis when GRP78 was knocked down with siRNA, but when it was overexpressed, the combination caused apoptosis that was exacerbated (Figure 5). We hypothesized that the GRP78 which was knocked down by siRNA and induced by coadministration was offset. However, the GRP78 overexpression was additive with the up-regulation by the coadministration of two drugs.

GRP78 is highly expressed in lung cancer [38], breast cancer [39], and prostate cancer [40], and has also been reported in colorectal cancer. As a major regulator of endoplasmic reticulum stress, GRP78 may be closely related to tumor glycolysis and tumor microenvironment. Some scholars believe that GRP78 may be an important link in tumor metabolic reprogramming and immune escape. Therefore, GRP78 is regarded as a potential drug target for cancer intervention [41]. Nizar et al. [42] performed immunohistochemical analysis on normal colon tissue and moderately differentiated (stage II), poorly differentiated (stage III), and metastatic colon cancer tissue to lung (stage IV). They found that the GRP78 immunoreactivity score (IRS) was 3, 26, 38, and 40, respectively, and the positive rates of tumor tissue were 78%, 89%, and 100%, respectively. Besides, the cleaved XBP1 mRNA was also higher expressed than that of normal tissue, indicating that UPR was constitutively activated in colon cancer tissue. In addition, inhibition of GRP78 in the CRC cell line increases the sensitivity to the DNA-targeted chemotherapeutic drugs, cisplatin and 5- fluorouracil. Michael et al. [43] collected 396 samples of CRC patients and detected the expression of GRP78 on tissue microarrays using IHC microarray technology. The results showed that the expression of GRP78 in the tumor was significantly higher than that in adjacent tissues (*P* < 0.0001), and was related to the degree of invasion (*P*=0.029) and stage (*P*=0.032). The increase in 5-year overall survival was associated with high expression of GRP78 (*P*=0.036). In vitro experiments showed that inhibition of GRP78 reduced the apoptosis of p53 wild-type CRC cells induced by 5-FU. Therefore, although GRP78 is highly expressed in some tumor tissues, in vitro experiments have shown that the interference of GRP78 has different effects on tumor cells. Whether GRP78 plays a role in pro-survival or death may depend on the state of cells. When cells were under stress from unfolded proteins, GRP78 conjugated with these proteins to prevent misfolding, thus playing a role in promoting cell survival [44]. However, under the stress of unfolded proteins, the membrane translocation of GRP78 might make it become a pro-apoptotic membrane receptor [45]. When liver cancer cells are treated with insulin-like growth factor-I (IGF-I), GRP78 redistributes from the endoplasmic reticulum (ER) to the cell surface and promotes its physical interaction with IGF-IR. The inhibition of cellular GRP78 membrane translocation by EGCG may be related to its interference with the physical interaction between IGF-I and GRP78 [46]. Consistent with the in vitro results, EGCG promoted the expression of GRP78 in the tumor tissue of HCT116 xenografts mice and eventually induced tumor cell apoptosis (Figure 6).

The relationship between ROS and endoplasmic reticulum stress (ERS) has been reported before, and ROS may be the cause or the result of ERS [47, 48]. However, it has also been reported that damage to molecular chaperones and proteasomal degradation pathways by certain factors may reduce intracellular ROS during ER stress, such as denatured heme globin aggregates [49], Deletion of the stress-inducible gene ERV29 necessary for ER-associated degradation (ERAD) [50]. The way in which EGCG exerts its antioxidant effect may involve the oxidation of its own phenolic group, and the activation of the transcription factor Nrf2. EGCG can also produce mitochondrial uncoupling, and slight uncoupling can attenuate mitochondrial ROS production [51]. The ER is highly sensitive to stress factors that alter cellular energy levels, calcium homeostasis, or redox state [52], and changes in redox homeostasis may be the main cause of EGCG-induced ER stress in CRC cells.

In conclusion, in the present study, we demonstrate the role of GRP78-mediated endoplasmic reticulum stress in the synergistic inhibition of colorectal cancer by Epigallocatechin-3-gallate and irinotecan. EGCG increases the intracellular accumulation of GRP78 and inhibits its cell membrane translocation, promotes the conversion of constitutive UPR to endoplasmic reticulum stress in colorectal cancer cells, and induces apoptosis. We identify that the bioactive substance EGCG has a good synergistic anti-colorectal cancer effect, and the combination of it with clinical chemotherapy drugs may be a new strategy to enhance anti-tumor efficacy and minimize drug side effects.

## Figures and Tables

**Table 1:** Sequences of the primers used in the real-time PCR.

Gene	Primer sequence (5′-3′)
GRP78	5′- CATCACGCCGTCCTATGTCG-3′
	5′- CGTCAAAGACCGTGTTCTCG-3′

GAPDH	5′-ATTTGGTCGTATTGGGCG-3′
	5′-CATGTAGTTGAGGTCAATGA-3′

## Data Availability

The data generated in the present study may be requested from the corresponding author.

## Abbreviations

ANOVA: One-way analysis of variance
CRC: Colorectal cancer
DCFH-DA: 2′, 7′-dichlorofluorescin diacetate
EGCG: Epigallocatechin-3-gallate
ERS: Endoplasmic reticulum stress
GRP78: Glucose regulatory protein 78 kDa
IRI: Irinotecan
JC-1: 5, 5′, 6, 6′-Tetrachloro-1, 1′, 3, 3′-tetraethyl-imidacarbocyanine iodide
MMP: Mitochondrial membrane potential
READ: Rectum adenocarcinoma
ROS: Reactive oxygen species
TMP: 3, 3′, 5, 5′- tetramethylbenzidine.

## References

[1] Bray F., Ferlay J., Soerjomataram I., Siegel R. L., Torre L. A., Jemal A. (2018). Global cancer statistics 2018: GLOBOCAN estimates of incidence and mortality worldwide for 36 cancers in 185 countries. *CA: A Cancer Journal for Clinicians*.

[2] National Health Commission of the People’s Republic of China (2020). Chinese protocol of Diagnosis and treatment of colorectal cancer (2020 edition). *Zhonghua wai ke za zhi [Chinese journal of surgery]*.

[3] Chiu Y. H., Hsu S. H., Hsu H. W. (2018). Human non-small cell lung cancer cells can be sensitized to camptothecin by modulating autophagy. *International Journal of Oncology*.

[4] Campbell J. M., Stephenson M. D., Bateman E., Peters M. D. J., Keefe D. M., Bowen J. M. (2017). Irinotecan-induced toxicity pharmacogenetics: an umbrella review of systematic reviews and meta-analyses. *The Pharmacogenomics Journal*.

[5] Abe S. K., Inoue M. (2020). Green tea and cancer and cardiometabolic diseases: a review of the current epidemiological evidence. *European Journal of Clinical Nutrition*.

[6] Almatroodi S. A., Almatroudi A., Khan A. A., Alhumaydhi F. A., Alsahli M. A., Rahmani A. H. (2020). Potential therapeutic targets of epigallocatechin gallate (EGCG), the most abundant catechin in green tea, and its role in the Therapy of various types of cancer. *Molecules*.

[7] He J., Xu L., Yang L., Wang X. (2018). Epigallocatechin gallate is the most effective catechin against antioxidant stress via Hydrogen Peroxide and radical scavenging activity. *Medical Science Monitor*.

[8] Gan R. Y., Li H. B., Sui Z. Q., Corke H. (2018). Absorption, metabolism, anti-cancer effect and molecular targets of epigallocatechin gallate (EGCG): an updated review. *Critical Reviews in Food Science and Nutrition*.

[9] Ding S., Xu S., Fang J., Jiang H. (2020). The Protective effect of polyphenols for colorectal cancer. *Frontiers in Immunology*.

[10] Wu W., Dong J., Gou H. (2021). EGCG synergizes the therapeutic effect of irinotecan through enhanced DNA damage in human colorectal cancer cells. *Journal of Cellular and Molecular Medicine*.

[11] Dicks N., Bohrer R. C., Gutierrez K., Michalak M., Agellon L. B., Bordignon V. (2017). Relief of endoplasmic reticulum stress enhances DNA damage repair and improves development of pre-implantation embryos. *PLoS One*.

[12] Yamamori T., Meike S., Nagane M., Yasui H., Inanami O. (2013). ER stress suppresses DNA double-strand break repair and sensitizes tumor cells to ionizing radiation by stimulating proteasomal degradation of Rad51. *FEBS Letters*.

[13] Oakes S. A. (2020). Endoplasmic reticulum stress signaling in cancer cells. *American Journal Of Pathology*.

[14] Kim C., Kim B. (2018). Anti-cancer Natural products and their bioactive compounds inducing ER stress-mediated apoptosis: a review. *Nutrients*.

[15] Karthikeyan B., Harini L., Krishnakumar V., Kannan V. R., Sundar K., Kathiresan T. (2017). Insights on the involvement of (-)-epigallocatechin gallate in ER stress-mediated apoptosis in age-related macular degeneration. *Apoptosis*.

[16] Ranzato E., Martinotti S., Magnelli V. (2012). Epigallocatechin-3-gallate induces mesothelioma cell death via H2 O2 -dependent T-type Ca2+ channel opening. *Journal of Cellular and Molecular Medicine*.

[17] Philippeos C., Hughes R. D., Dhawan A., Mitry R. R. (2012). Introduction to cell culture. *Methods in Molecular Biology*.

[18] Rio D. C., Ares M., Hannon G. J., Nilsen T. W. (2010). Purification of RNA using TRIzol (TRI reagent). *Cold Spring Harbour Protocols*.

[19] Chazotte B. (2011). Labeling mitochondria with JC-1. *Cold Spring Harbour Protocols*.

[20] Eruslanov E., Kusmartsev S. (2010). Identification of ROS using oxidized DCFDA and flow-cytometry. *Methods in Molecular Biology*.

[21] Martinotti S., Ranzato E., Burlando B. (2018). (−)- Epigallocatechin-3-gallate induces GRP78 accumulation in the ER and shifts mesothelioma constitutive UPR into proapoptotic ER stress. *Journal of Cellular Physiology*.

[22] Misra U. K., Gonzalez-Gronow M., Gawdi G., Hart J. P., Johnson C. E., Pizzo S. V. (2002). The role of Grp 78 in alpha 2-macroglobulin-induced signal transduction. Evidence from RNA interference that the low density lipoprotein receptor-related protein is associated with, but not necessary for, GRP 78-mediated signal transduction. *Journal of Biological Chemistry*.

[23] Gray P. C., Vale W. (2012). Cripto/GRP78 modulation of the TGF-*β* pathway in development and oncogenesis. *FEBS Letters*.

[24] Lourenço E. V., Roque-Barreira M. C. (2010). Immunoenzymatic quantitative analysis of antigens expressed on the cell surface (cell-ELISA). *Methods in Molecular Biology*.

[25] Sun X., Huo X., Luo T., Li M., Yin Y., Jiang Y. (2011). The anticancer flavonoid chrysin induces the unfolded protein response in hepatoma cells. *Journal of Cellular and Molecular Medicine*.

[26] Zhang S., Cao M., Fang F. (2020). The role of epigallocatechin-3-gallate in autophagy and endoplasmic reticulum stress (ERS)-Induced apoptosis of human diseases. *Medical Science Monitor*.

[27] Fang W., Hao L., Pei Z., Guo-jun J., Lin-yan M., Chao C. (2013). The inhibitory action of EGCG on the proliferation in the triple-negative breast cancer cell MDA-MB-231 and its mechanism. *Chinese Pharmacological Bulletin*.

[28] Das A., Banik N. L., Ray S. K. (2010). Flavonoids activated caspases for apoptosis in human glioblastoma T98G and U87MG cells but not in human normal astrocytes. *Cancer*.

[29] Filippi A., Picot T., Aanei C. M. (2018). Epigallocatechin-3-O-gallate alleviates the malignant phenotype in A-431 epidermoid and SK-BR-3 breast cancer cell lines. *International Journal of Food Sciences & Nutrition*.

[30] Panieri E., Santoro M. M. (2016). ROS homeostasis and metabolism: a dangerous liaison in cancer cells. *Cell Death & Disease*.

[31] de Sa Junior P. L., Camara D. A. D., Porcacchia A. S. (2017). The roles of ROS in cancer heterogeneity and Therapy. *Oxidative Medicine and Cellular Longevity*.

[32] Satoh M., Takemura Y., Hamada H., Sekido Y., Kubota S. (2013). EGCG induces human mesothelioma cell death by inducing reactive oxygen species and autophagy. *Cancer Cell International*.

[33] Tao L. (2015). The differential pro-oxidant effects of the tea (Camellia sinensis) catechin, (-)-epigallocatechin-3-gallate (EGCG). *The Context of Oral Cancer*.

[34] Okon I. S., Coughlan K. A., Zhang M., Wang Q., Zou M.-H. (2015). Gefitinib-mediated reactive oxygen specie (ROS) Instigates mitochondrial dysfunction and drug resistance in lung cancer cells. *Journal of Biological Chemistry*.

[35] Gutiérrez T., Simmen T. (2018). Endoplasmic reticulum chaperones tweak the mitochondrial calcium rheostat to control metabolism and cell death. *Cell Calcium*.

[36] Magyar J., Gamberucci A., Konta L. (2009). Endoplasmic reticulum stress underlying the pro-apoptotic effect of epigallocatechin gallate in mouse hepatoma cells. *The International Journal of Biochemistry & Cell Biology*.

[37] Hernandez I., Cohen M. (2022). Linking cell-surface GRP78 to cancer: from basic research to clinical value of GRP78 antibodies. *Cancer Letters*.

[38] Xia S., Duan W., Liu W., Zhang X., Wang Q. (2021). GRP78 in lung cancer. *Journal of Translational Medicine*.

[39] López-Muñoz E., Corres-Molina M., García-Hernández N. (2020). Correlation of the protein expression of GRP78 and BIK/NBK with prognostic markers in patients with breast cancer and neoadjuvant chemotherapy. *Journal of Obstetrics and Gynaecology*.

[40] Zhang X., Zhang Y., Lin F., Shi X., Xiang L., Li L. (2020). Shh overexpression is Correlated with GRP78 and AR expression in primary prostate cancer: Clinicopathological features and Outcomes in a Chinese Cohort. *Cancer Management and Research*.

[41] Madhavan S., Nagarajan S. (2020). GRP78 and next generation cancer hallmarks: an underexplored molecular target in cancer chemoprevention research. *Biochimie*.

[42] Mhaidat N. M., Alzoubi K. H., Khabour O. F., Banihani M. N., Al-Balas Q. A., Swaidan S. (2016). GRP78 regulates sensitivity of human colorectal cancer cells to DNA targeting agents. *Cytotechnology*.

[43] Thornton M., Aslam M. A., Tweedle E. M. (2013). The unfolded protein response regulator GRP78 is a novel predictive biomarker in colorectal cancer. *International Journal of Cancer*.

[44] Elfiky A. A., Baghdady A. M., Ali S. A., Ahmed M. I. (2020). GRP78 targeting: Hitting two birds with a stone. *Life Sciences*.

[45] Ge R., Kao C. (2019). Cell surface GRP78 as a death receptor and an anticancer drug target. *Cancers*.

[46] Yin Y., Chen C., Chen J. (2017). Cell surface GRP78 facilitates hepatoma cells proliferation and migration by activating IGF-IR. *Cellular Signalling*.

[47] Malhotra J. D., Kaufman R. J. (2007). Endoplasmic reticulum stress and oxidative stress: a vicious cycle or a double-edged sword?. *Antioxidants and Redox Signaling*.

[48] Bhandary B., Marahatta A., Kim H. R., Chae H. J. (2012). An involvement of oxidative stress in endoplasmic reticulum stress and its associated diseases. *International Journal of Molecular Sciences*.

[49] Suragani R. N. V. S., Zachariah R. S., Velazquez J. G. (2012). Heme-regulated eIF2*α* kinase activated Atf4 signaling pathway in oxidative stress and erythropoiesis. *Blood*.

[50] Haynes C. M., Titus E. A., Cooper A. A. (2004). Degradation of misfolded proteins prevents ER-derived oxidative stress and cell death. *Molecular Cell*.

[51] Mezera V., Endlicher R., Kucera O., Sobotka O., Drahota Z., Cervinkova Z. (2016). Effects of epigallocatechin gallate on Tert-ButylHydroperoxide-induced mitochondrial dysfunction in Rat liver mitochondria and Hepatocytes. *Oxidative Medicine and Cellular Longevity*.

[52] Glab J. A., Doerflinger M., Nedeva C. (2017). DR5 and caspase-8 are dispensable in ER stress-induced apoptosis. *Cell Death Differ*.

